# Contracted time and expanded space: The impact of circumnavigation on judgements of space and time

**DOI:** 10.1016/j.cognition.2017.06.004

**Published:** 2017-09

**Authors:** Iva K. Brunec, Amir-Homayoun Javadi, Fiona E.L. Zisch, Hugo J. Spiers

**Affiliations:** a Institute of Behavioural Neuroscience, Department of Experimental Psychology, University College London, London, UK; b Department of Psychology, University of Toronto, Toronto, Canada; c Rotman Research Institute, Toronto, Canada; d School of Psychology, University of Kent, Canterbury, UK; e The Bartlett School of Architecture, University College London, London, UK

**Keywords:** Spatial navigation, Time estimation, Spatial boundaries, Grid cells, Temporal memory

## Abstract

The ability to estimate distance and time to spatial goals is fundamental for survival. In cases where a region of space must be navigated around to reach a location (circumnavigation), the distance along the path is greater than the straight-line Euclidean distance. To explore how such circumnavigation impacts on estimates of distance and time, we tested participants on their ability to estimate travel time and Euclidean distance to learned destinations in a virtual town. Estimates for approximately linear routes were compared with estimates for routes requiring circumnavigation. For all routes, travel times were significantly underestimated, and Euclidean distances overestimated. For routes requiring circumnavigation, travel time was further underestimated and the Euclidean distance further overestimated. Thus, circumnavigation appears to enhance existing biases in representations of travel time and distance.

## Highlights

• Certain locations in the world can only be reached by circumnavigating obstacles.
• All travel times are underestimated and all distances are overestimated.
• Travel times are further compressed when circumnavigation is required.
• Distances are further expanded when circumnavigation is required.
• Temporal and spatial cognitive biases may be dissociable.

## 1 Introduction

Knowing how far away a destination is or how quickly one can travel there can be important for survival and shapes our daily lives. Ideally, our estimates of distance and time would be accurate, but often they are systematically distorted by many factors, such as the number of turns required, density of structures in the environment, and familiarity with the environment (Arnold et al., 2016, Bonasia et al., 2015, Briggs, 1973, Jafarpour and Spiers, 2016, Sadalla and Magel, 1980, Saisa et al., 1986, Thorndyke, 1981).

In some situations, it can be necessary to circumnavigate an obstacle in the environment to reach a location. Navigating to a goal in the world and returning home requires knowledge of the environmental geometry and, frequently, the ability to circumnavigate obstacles while keeping track of the goal’s location (McNaughton et al., 2006, Mittelstaedt and Mittelstaedt, 1980). Such circumnavigation, however, introduces disparities between path distance and straight-line (Euclidean) distance to the goal. Recent neuroimaging research has shown that medial temporal lobe (MTL) regions track the distance to the goal during navigation (Balaguer et al., 2016, Chrastil et al., 2015, Morgan et al., 2011, Sherrill et al., 2013, Spiers and Maguire, 2007, Viard et al., 2011), where activity in the entorhinal region correlated with Euclidean distance and activity in the posterior hippocampus correlated with the path distance (Howard et al., 2014). At decision points, hippocampal activity was related to both how close the goal was and the egocentric direction to it (Howard et al., 2014). Activity was maximal when the goal was close and directly ahead and low when the goal was along a path curved away from the current heading and far away (Howard et al., 2014). Thus, it seems possible that the geometry of the path to the goal may systematically impact on how the brain represents space. However, there has been little investigation of how the geometry of a path impacts on the internal representation of the route or the spatial relationship to the goal, despite the suggestion that environmental geometry provides a crucial orientation cue to both animals and humans (Cheng, 1986, Cheng and Newcombe, 2005, Gallistel, 1990). However, it remains unknown if the environmental geometry of a path (curvature) has a significant impact on estimates of the distance or the time estimated to travel to goals.

**Fig. 1:**
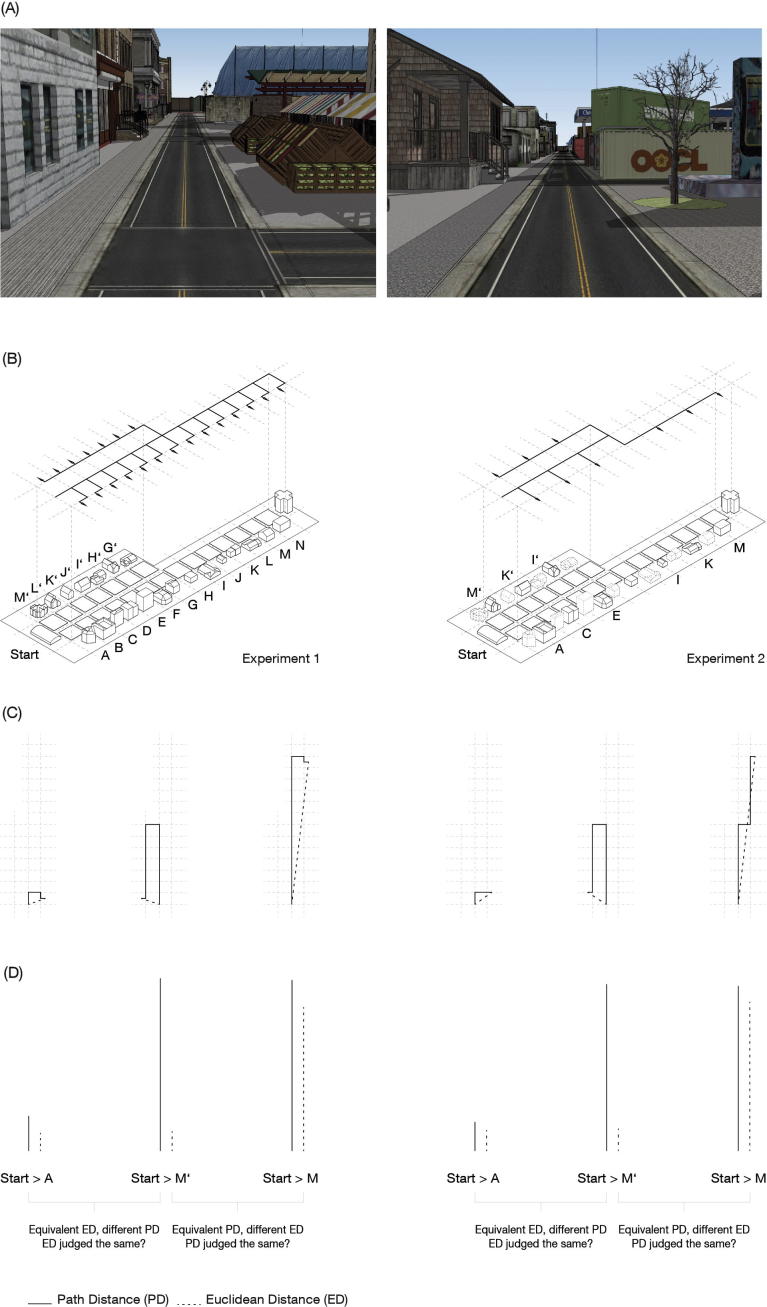
Virtual reality town used in the experiments. (A) Example screenshots of views participants would have experienced in the task. (B) Overhead schematic views of the environmental layout for Experiments 1 and 2. The starting location (Pizzeria) is marked. Lines with arrows indicate possible paths from the starting point to learned goal locations. In Experiment 2, the environment was identical, but participants only delivered to 9 locations. The laterality of the elongated section was counterbalanced across participants – it was located on the left hand side for half of the participants and on the right hand side for the other half in each experiment. (C) A one-way system of routes was constructed to create pairs of routes with equal PD but different ED, all with equal numbers of turns. In Experiment 1, each goal location was in the middle of each road segment and in Experiment 2, it was at the junction. To reach goals on L-shaped routes in Experiment 1, participants travelled along the main road until they believed they had reached the correct turning point. In Experiment 2, participants were required to make a turn as soon as they reached the elongated section of the environment, therefore controlling for exposure to all locations with matched PD in Experiment 2. (D) Examples of pairs of routes with equal path distance but different Euclidean distance and vice versa.

Here, we used verbal judgments to measure biases in the estimates of travel time and Euclidean distance on routes to goals that either matched in path distance but differed in Euclidean distance, or matched in Euclidean distance but differed in path distance. We created a virtual reality (VR) environment to control for prior experience, curvature, direction, and angle to goal during navigation (Fig. 1). In two experiments, participants travelled to different numbers of locations in the environment.

We predicted that on U-shaped routes, the goal might be perceived as farther away because the travel time would lead to an impression of it being conceptually farther away. We considered that time estimation might plausibly decrease or lengthen with the curvature.

## 2 Experiment 1

### 2.1 Methods

Twenty-three participants took part in Experiment 1 (15 females). Their age range was 18–30 years (mean 22.2 years), all were right-handed, and none reported any history of psychiatric or neurological disorders. All participants gave their informed consent. This research was approved by the ethics committee at University College London.

Participants were instructed that their task would be to deliver pizzas to various locations in the virtual town. A one-way system of routes was constructed to create pairs of routes with equal Euclidean distance but different path distance (Fig. 1). The virtual town was built to a consistent scale so the size of buildings and blocks was representative of real-world objects/buildings and could be used to infer distances when making estimates. There were 21 locations. The driving speed was set to approximately 35 km/h. Participants were first led through the town by pressing arrow keys corresponding to green arrows displayed on the screen. The order of the routes was randomized, but in every three trials, one route was sampled from each part of the environment (A-E, I-M, I′-M′). In this drive-through, they were forced to turn toward each goal location before they could continue, ensuring exposure to all goal locations prior to delivering to them. After the drive-through, the participants were instructed to find the shortest possible route for each goal location contingent on the one-way road layout from the pizzeria as their starting point. Their goal was displayed in the upper right hand corner throughout the search. After each delivery, they were teleported to the starting point and given a new goal.

The participants were then instructed that their task and the environment would remain the same but they would additionally have to estimate the duration of each delivery prior to each journey (*time estimation*) and then to reach each goal using the shortest, most direct route possible. A probe window appeared at the start of each journey asking participants to type in the number of seconds they thought the journey would take. They again navigated to each location 3 times. After completing all navigation trials, participants were asked to estimate straight-line distances (*Euclidean distance estimation*) to each of the goals shown to them one at a time without any background or surrounding buildings in a randomized order.

### 2.2 Results

The travelled time was subtracted from estimates to yield a *bias score* for the degree of under- or overestimation observed on each trial. These were averaged across the three visits to each location. Similarly, bias scores for distance estimates were calculated by subtracting actual from estimated Euclidean distance (ED). All suboptimal journeys (any path other than the shortest possible) were excluded from the analysis, excluding 3.73% of trials on U-shaped and 20.6% on L-shaped routes. This discrepancy is due to more frequent exposure to locations along the U-shaped routes. For example, participants had to travel past G’ every time they delivered to any location along U-shaped routes, while this was not the case for L-shaped routes (see Fig. 1). This issue was resolved in Experiment 2. The distribution of errors can be found in Supplementary Materials (Fig. S1).

Participants’ mean travel time on L-shaped routes was 28.7 s (SD = 1.65) and the mean estimated time was 22.2 s (SD = 8.58). The mean travel time on U-shaped routes was 36.9 s (SD = 1.92) and estimated time was 26.9 s (SD = 8.19). In this experiment, travel times were significantly longer on U-shaped routes than L-shaped routes with matched PD (*t*_(22)_ = 9.84, p < 0.001; this is addressed and resolved in Experiment 2). This is due to a larger number of keypresses required each time participants travelled to locations on U-shaped routes, which was not the case on the L-shaped routes, where participants remained on the main road until they decided to make a turn (see Fig. 1). Participants’ estimates were then expressed as a proportion of actual travel times. The average proportion on L-shaped routes was 0.78 (SD = 0.25), and on U-shaped routes, it was 0.73 (SD = 0.21).

We then fitted two individual 2-level linear mixed-effects models to predict (1) bias and (2) proportion in time and ED estimates, averaged across the three repetitions. Participants were entered as a random factor. We compared only routes with matched PD for time estimates (G-M & G′-M′) and routes with matched ED for distance estimates (A-G & M′-G′). Prior to analysis, continuous independent variables (travelled time, Euclidean distance) were centred by subtracting the mean from each parameter, as per standard procedure in multi-level modelling. The strength of such linear generalised multi-level modelling is increased statistical power (Mathieu & Chen, 2011), as the inclusion of individual trials for each participant accounts for the maximal amount of variance in the dataset as the linear predictor contains random effects (in our case, participants) in addition to the fixed effects. The statistics are reported in Table 1.

**Table 1:** Results of the ANOVA run on the linear mixed model output.

		Route type	PD	Route type × PD	R^2^c
*Time estimates*, *matched PD*					
Exp. 1	Bias score	*F*(*1*, *286*) = 15.83, *P* < 0.001	*F*(*1*, *286*) = 37.72, *P* < 0.001	*F*(*1*, *286*) = 2.95, *P* = 0.087	74.4%
	Proportion	*F*(*1*, *286*) = 9.73, *P* = 0.002	*F*(*1*, *286*) = 2.50, *P* = 0.115	*F*(*1*, *286*) = 1.07, *P* = 0.302	75.9%

Exp. 2	Bias score	*F*(*1*, *82*) = 7.98, *P* = 0.006	*F*(*1*, *82*) = 1.09, *P* = 0.299	*F*(*1*, *82*) = 0.004, *P* = 0.948	90.1%
	Proportion	*F*(*1*, *82*) = 11.44, *P* = 0.001	*F*(*1*, *82*) = 7.98, *P* = 0.006	*F*(*1*, *82*) = 0.305, *P* = 0.582	93.0%

*Distance estimates*, *matched ED*					
Exp. 1	Bias score	*F*(*1*, *296*) = 5.23, *P* = 0.023	*F*(*1*, *296*) = 0.122, *P* = 0.728	*F*(*1*, *296*) = 21.62, *P* < 0.001	72.3%
	Proportion	*F*(*1*, *296*) = 10.97, *P* = 0.001	*F*(*1*, *296*) = 0.559, *P* = 0.456	*F*(*1*, *296*) = 0.695, *P* = 0.405	79.7%

Exp. 2	Bias score	*F*(*1*, *97*) = 11.17, *P* = 0.001	*F*(*1*, *97*) = 0.497, *P* = 0.483	*F*(*1*, *97*) = 11.11, *P* = 0.001	78.3%
	Proportion	*F*(*1*, *97*) = 17.82, *P* < 0.001	*F*(*1*, *97*) = 8.26, *P* < 0.001	*F*(*1*, *97*) = 15.43, *P* < 0.001	76.9%

For Experiment 1, routes with matched path distance (PD) include goals G-M on L-shaped routes, and goals M′-G′ on U-shaped routes. Routes with matched ED include goals A-G on L-shaped routes, and goals M′-G′ on U-shaped routes. For Experiment 2, routes with matched PD include goals I-M on L-shaped routes, and goals M′-I′ on U-shaped routes. Routes with matched ED include goals A-E on L-shaped routes, and goals M′-I′ on U-shaped routes. The residual degrees of freedom are reflective of the number of trials included in each model. The effect sizes are expressed as conditional R^2^ values for each model (R^2^c), which describe the proportion of variance accounted for by the fixed and random factors in the model (Nakagawa & Schielzeth, 2013). Parameter estimates and 95% confidence intervals are reported in Table S1 in the Supplementary Materials.

**Fig. 2:**
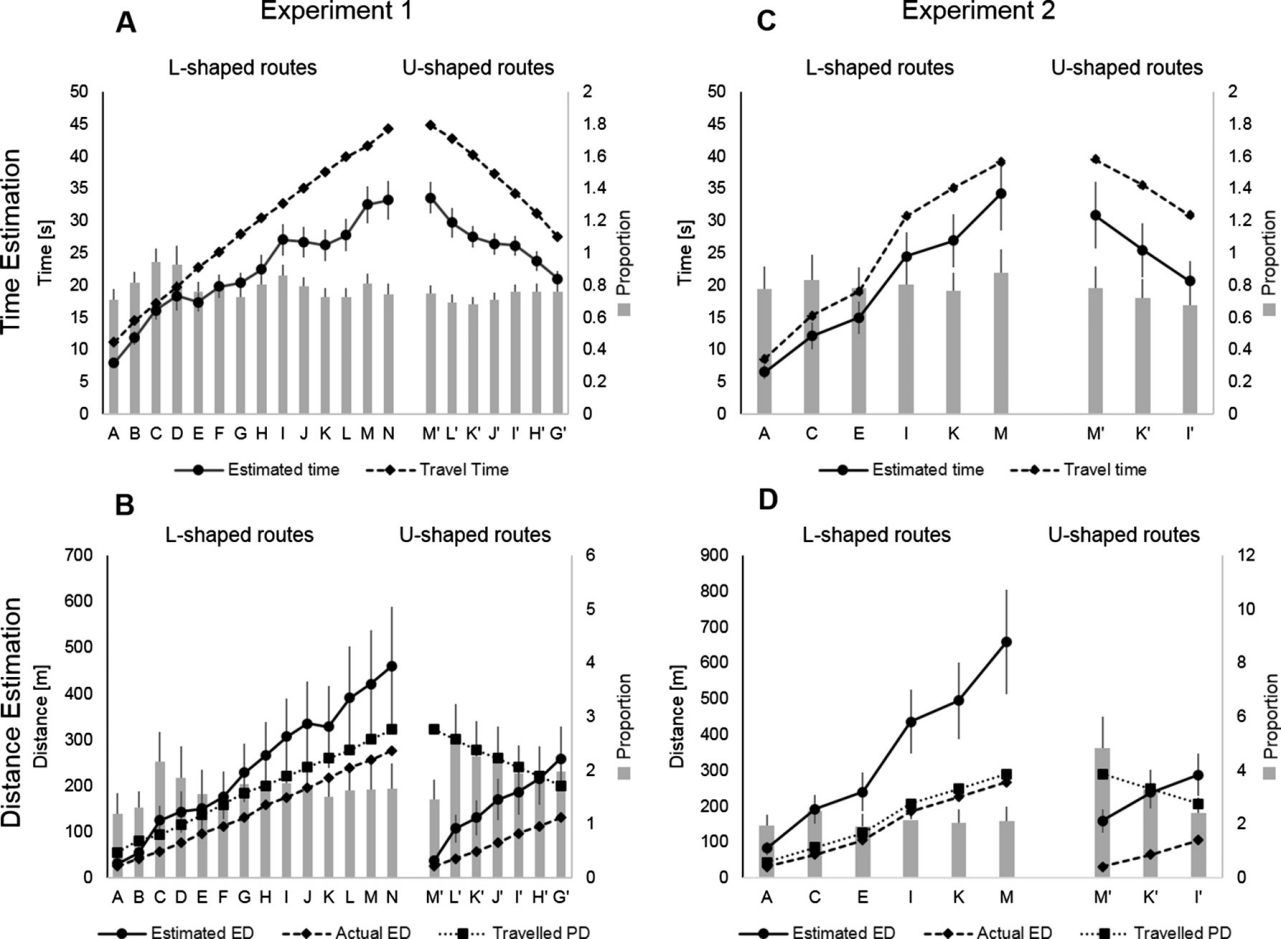
Time and Distance Estimates. (A) Estimated and actual travel times on L-shaped (A-N) and U-shaped (M′-G′) routes in Experiment 1. Grey bars express proportions of estimated/actual travel time. There was a significant effect of route type, such that underestimation was significantly greater for locations closer in terms of ED when PD was matched. (B) Estimated and actual Euclidean distances on L-shaped and U-shaped routes in Experiment 1. Grey bars express proportions of estimated/actual distances. (C) Estimated and actual travel times on L-shaped and U-shaped routes in Experiment 2. As in Experiment 1, underestimation was greater on U-shaped, relative to L-shaped routes. (D) Estimated and actual distances in Experiment 2. Distances were significantly overestimated – by a factor of 2 on L-shaped routes and 3 on U-shaped routes. All error bars represent standard error. Note that the proportion bars represent relative proportions and thus do not directly correspond to the difference between the two lines.

We found that estimated travel time was significantly underestimated as the travel time increased and that this underestimation was significantly greater on U-shaped routes (Table 1, Fig. 2A). When analysis focused on the proportion of the estimate relative to the correct travel time, there was a significant main effect of route type, but no significant main effect of PD, suggesting that while underestimation was greater on U-shaped routes overall, these proportions did not significantly change as a function of the actual distance travelled (reflected by the grey bars in Fig. 2A).

The same analyses were applied to ED estimates. In contrast to time estimates, distances were consistently and increasingly overestimated (Fig. 2B). The mean estimated ED on U-shaped routes (158.4 m; SD = 196.2) was significantly greater than the mean estimated ED on L-shaped routes (130.0 m; SD = 165.7): *t*_(22)_ = 2.59, *P* = 0.017. Bias in ED estimates was modelled as a function of route type and PD for locations with matched ED. There was a significant main effect of route type (*P* = 0.023) and a significant interaction between route type and PD, suggesting that bias increased as a function of path distance and that this bias was increased for locations on U-shaped routes (Fig. 2A).

Participants’ estimates were again calculated as proportions of actual Euclidean distance. On L-shaped routes, participants overestimated distances by a factor of 1.63 (SD = 2.05) and on U-shaped routes by 2.04 (SD = 2.45), indicating that locations on U-shaped routes were perceived to be on average twice as far away as they were in reality. The main effect of route type was again significant, but the route type x PD interaction was not (Fig. 2B).

In Experiment 2, we aimed to address the issues of the greater number of suboptimal routes chosen for L-shaped routes than U-shaped routes and different mean travel times to locations on U-shaped and L-shaped routes. The key differences were: (1) all goal locations were moved to intersections to prevent the difference in travel times to locations along L-shaped and U-shaped routes. This made the number of key presses equal and controlled travel times. (2) To control for goal exposure, number of turns, and attempt to achieve equivalent rates of optimal route taking, a turn was added to the L-shaped routes. Participants therefore travelled past the goal locations on U-shaped and L-shaped routes with matched PD (see Fig. 1). For the sake of clarity, routes with proportional Euclidean and path distances will still be referred to as L-shaped. (3) Critically, time and distance estimates were made in a counterbalanced order after all navigation trials were complete. This way, participants were prevented from experiencing the routes again and counting.

## 3 Experiment 2

### 3.1 Methods

Twenty participants took part in Experiment 2 (12 female, 8 male). Their mean age was 23.4 years (SD = 2.70). All participants were right handed and had normal or corrected-to-normal vision and none reported any history of psychiatric or neurological problems. Participants provided their informed consent. The study was approved by the ethics committee at University College London.

In order for the participants to rapidly learn the one-way system of roads and increase the rate of optimal routes, the experimenter verbally guided participants to each of the locations three times along the optimal route in the training phase. The order of locations was randomized in the same manner as in Experiment 1. Each participant received the same instructions for each location in the environment. After they were guided to each location three times, the participants navigated to each location independently three more times. These trials provided travel time measures for the optimal routes. Afterwards, participants were asked to estimate travel time and Euclidean distance to each location three times in a pseudorandomized order, such that each three consecutive images were always from the three respective parts of the virtual town. This was done to prevent participants from basing their consecutive estimates on locations near one another.

### 3.2 Results

The percentage of optimally taken routes was 88.6% on L-shaped routes and 88.9% on U-shaped routes. All participants took the optimal route to each location at least once, meaning no routes were excluded at retrieval. There was no significant difference between travel times on L-shaped and U-shaped routes with equal path distance (travel time M on L-shaped routes = 35.13, SD = 4.43; U-shaped routes M = 35.4, SD = 4.35; *F* < 1). Three participants (1 M, 2 F) were removed from subsequent analyses as their mean estimates fell more than 2 SDs above the group mean.

We conducted the same analysis as used for Experiment 1 to allow for direct comparison between the two experiments (see Table 1). Time estimation bias for pairs of L-shaped and U-shaped routes with matched PD was modelled as a function of route type and PD. We again only compared routes with matched PD for time estimates (I-M & I′-M′) and routes with matched ED for distance estimates (A-E & M′-I′). The mean time estimate on L-shaped routes was 28.51 s (SD = 18.90) and on U-shaped routes with matched PD it was 25.65 s (SD = 17.41), again reflecting underestimation on all routes. There was a main effect of route type (p = 0.006), but no significant main effect of PD (Fig. 2C). We therefore replicate the finding from Experiment 1 indicating that U-shaped routes are significantly more underestimated than L-shaped ones. In contrast to Experiment 1, we do not observe an increase in underestimation as a function of PD. This may be because fewer locations were sampled in Experiment 2, and this lower memory load allowed participants to create more stable representations. Another possibility is that this is the result of controlling for exposure. In Experiment 2, but not Experiment 1, participants travelled past all the locations in the elongated section on L-shaped routes (Fig. 1B), which may have decreased this bias.

We then calculated the proportion of participants’ time estimates relative to actual travel times. The proportion on L-shaped routes was 0.81 (SD = 0.55) and on U-shaped routes, it was 0.73 (SD = 0.47). We again find a significant main effect of route type, but additionally we also find a significant main effect of PD, suggesting that estimation bias increased as a function of PD (Fig. 2C).

The mean distance estimate for locations on L-shaped routes was 171.7 m (SD = 188.0) and 288.4 m for U-shaped routes (SD = 213.9). Bias in distance estimates was modelled as a function of PD and route type for locations matched in ED. We found a main effect of route type and a significant PD × route type interaction, suggesting that overestimation was greater on U-shaped routes and this appeared to be modulated by the PD to each of the goal locations (Fig. 2D).

Participants’ distance estimates were then expressed as the proportion of actual distances. On L-shaped routes the proportion was 2.02 (SD = 2.07) and on U-shaped routes it was 3.0 (SD = 2.81). There was a significant main effect of route type, as well as a significant main effect of PD and a significant interaction, indicating that locations on U-shaped routes were perceived to be farther away along a straight line (ED) and that this was modulated by the PD to each of the locations (Fig. 2D).

**Fig. 3:**
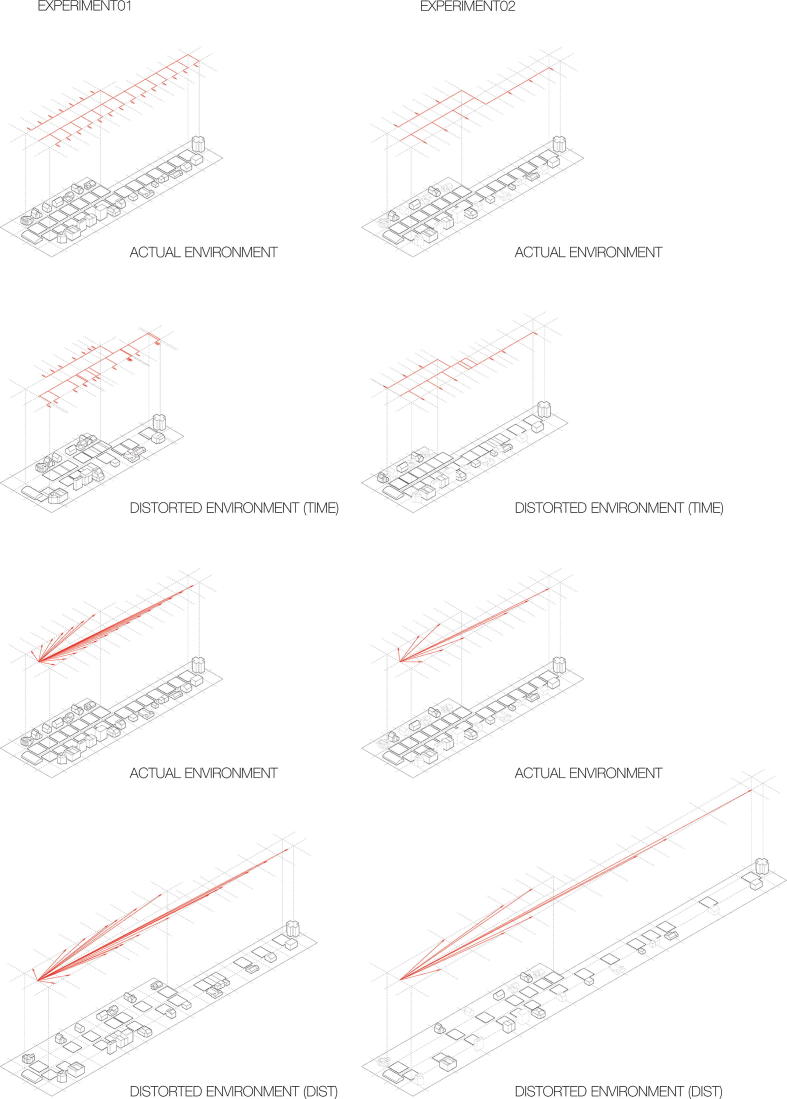
Visualisation of the results. Relative expansions and compressions specific to each location reflect the pronounced increase in temporal underestimation and distance overestimation on U-shaped routes. In Experiment 2, goal locations depicted with dotted lines were never delivered to, but were shown in the environment. In these plots, we placed those locations in the middle of each section between the appropriately scaled locations surrounding them.

We replicate our main finding of greater temporal compression and distance expansion on U-shaped routes, while equating the number of suboptimal routes taken and controlling for travel time to locations on U-shaped routes, which were the issues identified in Experiment 1. This effect therefore appears to be robust, regardless of the number of routes and whether estimates are prospective (Experiment 1) or retrospective (Experiment 2). We visualised the bias participants showed in both experiments by scaling the environment corresponding to their bias (Fig. 3).

## 4 Discussion

We provide novel evidence that circumnavigating part of an environment leads to a contraction in the estimated travel time and expansion of the estimated Euclidean distance to locations on the route. These biases occur when the number of turns, exposure to the environment, and travel time are matched. They also occur in the context of a general tendency to underestimate travel time and overestimate Euclidean distance in more linear routes.

Participants underestimated travel time for all routes, consistent with previous research showing that humans tend to underestimate travel times (Arnold et al., 2016, Bonasia et al., 2015, Jafarpour and Spiers, 2016, Ziemer et al., 2009). Based on evidence from two prior studies, we would have expected distance to also be underestimated (Knapp and Loomis, 2004, Thompson et al., 2004). This was not the case in our study. In our near linear L-shaped routes, which made minimal demand on circumnavigation, we found that the farther away the goal, the greater the Euclidean distance overestimation, and the greater the time underestimation. This is not consistent with time and space estimates arising from a unified internal map of the space and being processed in a unified manner to derive estimates. If this was so, both time and distance would be underestimated or overestimated. This finding is consistent with recent evidence indicating that properties of the environment can lead to overestimates in distance and underestimates in travel time (Jafarpour & Spiers, 2016). While more study is needed to explore this apparent disparity in time and distance estimates, these studies together suggest a base-level dissociation between cognitive distortions in the temporal and spatial domains.

In contrast to L-shaped routes, the added overestimation in Euclidean distance and extra travel time compression for the locations circumnavigated to on the U-shaped routes are consistent with a unified adjustment in mapping of locations. This is because when a location is judged to be farther away in Euclidean distance, it logically should have a shorter path along the U-shaped route to reach it, thus a shorter travel time. Thus, the impact of having to circumnavigate a space appears to have a consistent effect on time and space.

In our experiments, we focused on how estimates of time and distance are distorted when the goal location is hidden to help understand how time and distance may be represented during wayfinding. It is likely that if the goal locations and paths to them had been fully visible, estimates to goal locations would have been less distorted. Past research indicates that on continuously textured plane, estimates of distance are relatively accurate. However, once discontinuities in texture are present, distances tend to be overestimated (Sinai, Ooi, & He, 1998). More study is needed to investigate whether goal visibility differentially influences distance and duration judgements. Spatial boundaries also likely affect the estimates of distance and time. Previous research has shown that spatial boundaries segment the incoming flow of information encoded into memory (Horner et al., 2016, Kurby and Zacks, 2008, Pettijohn et al., 2016, Radvansky and Copeland, 2006). It is possible that circumnavigation on U-shaped routes resulted in a perceived shift in context, making the more pronounced compression of time estimates and expansion of distance estimates context-specific. The level of exposure to each path in the environment might have also contributed to biases observed here. The present study design necessitated the traversal of the main path to reach all locations, meaning that participants passed paths leading to locations A-E on the way to all goals. While these locations could not be seen, the more frequent experience along the main path, relative to other paths, may have contributed to some of the observed biases. In future research, it will be useful to explore visibility, exposure, texture properties, and boundary properties to better understand distortions in estimates of distance and time.

At a neural level, entorhinal grid cells are thought to code the distance travelled by a read-out of the number of grid-fields traversed during navigation (Bush et al., 2015, Moser et al., 2008), which may be the basis for estimates of the distance to goals (Jafarpour & Spiers, 2016). Recent research recording from grid cells in rodents has indicated that the geometry of an environment can distort the internal representation of space, such that grid cell firing patterns in a square box became rotated or compressed in a specific direction when the environment’s geometry was distorted to form a trapezoid (Krupic et al., 2015, Stensola et al., 2015). Consistent with this, errors by humans solving a path integration task in a VR environment were consistent with predictions from a model using rodent grid cell firing properties (Chen, He, Kelly, Fiete, & McNamara, 2015). Given this evidence that environmental geometry can distort grid field spacing (Krupic et al., 2015, Stensola et al., 2015), it would be interesting to examine whether grid field spacing is distorted by circumnavigation in a manner consistent with the biased estimates we observed.

In sum, our study reveals an expansion in the subjective distance and contraction of the subjective travel time to the goal when path curvature increases due to circumnavigation. Our results have implications for fMRI studies that have explored how distance to the goal is coded in the MTL (Spiers and Barry, 2015), as we show that path curvature can lead to substantial differences in subjective distance, which could lead to categorical differences in which locations are subjectively experienced as being closer.

## Appendix A Supplementary material

Supplementary data 1

Supplementary Data S2 This is open data under the CC BY license (http://creativecommons.org/licenses/by/4.0/).
