# Supplementary material for: Contracted time and expanded space: The impact of circumnavigation on judgements of space and time
Source: Cognition. 2017 Sep;166:425–32. doi: 10.1016/j.cognition.2017.06.004 (PMC5495988; doi:10.1016/j.cognition.2017.06.004)
Supplement: Supplementary data 1 [file mmc1.docx]

**Supplementary Materials**

**Distribution of errors – suboptimal routes taken in Experiments 1 and 2**

In Experiment 1, suboptimal paths comprised 3.73% of trials on U-shaped and 20.6% on L-shaped routes.

In Experiment 2, suboptimal paths comprised 11.1% of trials on U-shaped routes and 11.4% on L-shaped routes.

The plots below represent the total error counts for each location (combined for all participants).


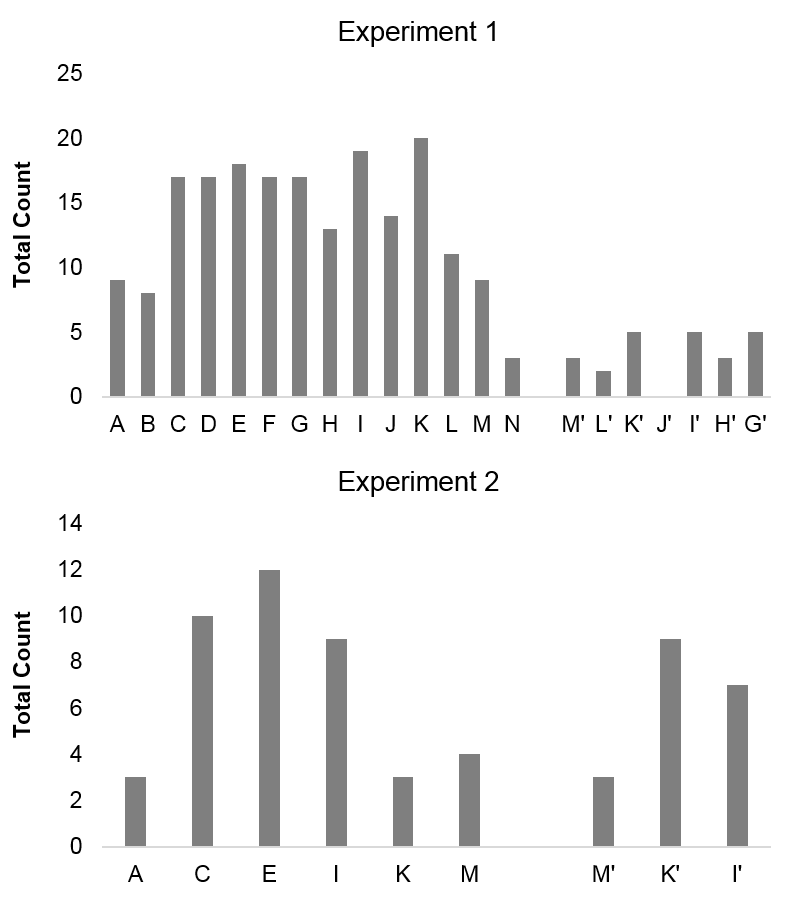


**Figure S1: Total numbers of suboptimal trials in Experiments 1 and 2.**

Examining these suboptimal route counts indicates that the locations at the two far ends of each path were most likely to be reached via an optimal route, whereas those in the middle of each section tended to be associated with more errors.

**Parameter estimates and 95% confidence intervals in the two experiments (effect size)**

**Table S1: Parameter estimates and 95% confidence intervals in square brackets for individual effects and interaction terms.**

| Time estimates, matched PD | | | | |
| --- | --- | --- | --- | --- |
|  |  | Route type | PD | Route type × PD |
| Exp. 1 | bias score | -3.569, [-10.24, 3.100] | -0.054, [-0.073, -0.035] | 0.024, [-0.003, 0.051] |
|  | proportion | -0.047, [-0.221, 0.128] | -0.000, [-0.001, 0.000] | 0.000, [-0.000, 0.001] |
| Exp. 2 | bias score | 3.698, [-11.43, 18.83] | 0.019, [-0.028, 0.066] | -0.002, [-0.069, 0.064] |
|  | proportion | 0.190, [-0.167, 0.143] | 0.001, [0.000, 0.002] | -0.000, [-0.002, 0.001] |
| Distance estimates, matched ED | | | | |
|  |  | Route type | PD | Route type × PD |
| Exp. 1 | bias score | -298.23, [-418.08, -178.93.734] | -0.823, [-1.266, -0.381] | 1.431, [0.828, 2.035] |
|  | proportion | -0.751, [-1.961, 0.458] | -0.000, [-0.005, 0.004] | 0.003, [-0.004, 0.009] |
| Exp. 2 | bias score | -290.98, [-462.85, -119.11] | -0.667, [-1.369, 0.036] | 1.690, [0.697, 2.684] |
|  | proportion | 5.700, [2.811, 8.588] | 0.029, [0.017, 0.041] | -0.033, [-0.050, -0.017] |
